# Longer latency of sensory response to intravenous odor injection predicts olfactory neural disorder

**DOI:** 10.1038/srep35361

**Published:** 2016-10-13

**Authors:** Shu Kikuta, Yu Matsumoto, Akihito Kuboki, Tsuguhisa Nakayama, Daiya Asaka, Nobuyoshi Otori, Hiromi Kojima, Takashi Sakamoto, Kashio Akinori, Kaori Kanaya, Rumi Ueha, Ryoji Kagoya, Hironobu Nishijima, Makiko Toma-Hirano, Yayoi Kikkawa, Kenji Kondo, Koichi Tsunoda, Tempei Miyaji, Takuhiro Yamaguchi, Kazunori Kataoka, Kensaku Mori, Tatsuya Yamasoba

**Affiliations:** 1 Departments of Otolaryngology, Graduate School of Medicine, University of Tokyo, 7-3-1 Hongo, Bunkyo-ku, Tokyo 113-8655, Japan; 2 Departments of Physiology, Graduate School of Medicine, University of Tokyo, 7-3-1 Hongo, Bunkyo-ku, Tokyo 113-8655, Japan; 3 Department of Materials Engineering, Graduate School of Engineering, University of Tokyo, 7-3-1, Hongo, Bunkyo-Ku, Tokyo, 113-8656, Japan; 4 Department of Otolaryngology, Jikei University School of Medicine, Tokyo, 3-25-8 Nishishinbashi, Minato-ku, Tokyo, 105-8461, Japan; 5 Department of Artificial Organs and Medical Device Creation, National Institute of Sensory Organs, Tokyo Medical Center, National Hospital Organization, Meguro-ku, Tokyo, 152-8902, Japan; 6 Departments of Clinical trial data management, Graduate School of Medicine, University of Tokyo, 7-3-1 Hongo, Bunkyo-ku, Tokyo 113-8655, Japan

## Abstract

A near loss of smell may result from conductive and/or neural olfactory disorders. However, an olfactory test to selectively detect neural disorders has not been established. We investigated whether onset latency of sensory response to intravenous odor injection can detect neural disorders in humans and mice. We showed that longer preoperative onset latency of odor recognition to intravenous odor in patients with chronic rhinosinusitis predicted worse recovery of olfactory symptoms following sinus surgery. The onset latency of the olfactory sensory neuron (OSN) response to intravenous odor using synaptopHluorin signals from OSN axon terminals was delayed in mice with reduced numbers of OSNs (neural disorder) but not with increased mucus or blocked orthonasal pathways (conductive disorders). Moreover, the increase in onset latency correlated with the decrease in mature OSN numbers. Longer onset latency to intravenous odor injection is a useful biomarker for presence and severity of olfactory disorders with neural etiology.

Sensory impairments (e.g., visual impairment, hearing loss, or olfactory deficiency) may be induced by conductive disorders, in which stimuli fail to reach sensory neurons, or by direct injury to sensory neurons. In some cases, appropriate functional tests have been developed to detect lesion sites and severity of sensory neuron loss^1^,^2^,^3^. In the visual system, combinations of electrophysiological tests such as electroretinograms and visually evoked potentials help to determine whether retinal neurons are involved in visual dysfunction. Similarly, bone conduction in the pure tone audiometry test is key to determine whether cochlear neurons are intact. However, for the olfactory system, an equivalent clinical test has not been established.

Approximately 20% of the population has some form of olfactory disorder, mainly caused by decreased olfactory sensory neuron (OSN) response to odor inhalation^4^,^5^,^6^,^7^,^8^,^9^. Loss of the sense of smell diminishes the emotional impact of food and has consequences for all aspects of life. Thus, appropriate olfactory function assessment tests are required to determine the etiology for selecting appropriate therapies, as well as to predict prognosis.

Many psychophysical assessments are used to evaluate olfactory disorders, and most are based on odor stimulation via either orthonasal or retronasal pathways (Fig. 1a). Orthonasal tests are the most popular. These tests involve OSN stimulation by sniffing odors through the orthonasal pathway^10^,^11^,^12^,^13^,^14^,^15^,^16^,^17^,^18^,^19^,^20^,^21^,^22^. Alternatively, in retronasal tests, OSNs are stimulated through the retronasal pathway (Fig. 1a)^22^,^23^,^24^,^25^,^26^,^27^. In the intravenous olfactory (IVO) test, which is considered a retronasal olfactory test, OSNs are stimulated by the intravenously administered odorant prosultiamine (PST). This test is widely used in Japan (Fig. 1b)^22^,^27^,^28^, but has received substantially less attention from sensory physiologists than orthonasal olfactory tests. However, it is advantageous compared to orthonasal tests because intravenously administered odors are instantaneously and abundantly released into a relatively enclosed cavity within the respiratory tract (nose, trachea, and alveolus), resulting in stronger OSN stimulation compared with that in orthonasal tests^22^,^29^. Two parameters of the response in the IVO test, latency of odor recognition onset from the time of PST injection and duration between odor recognition onset and disappearance, are measured to determine olfactory loss (Fig. 1b); however, it is unclear whether these two parameters are differentially altered by specific pathological conditions, such as loss of OSNs or reduced odorant conduction.

In the present study, the onset latency of odor detection in the pre-operative IVO test was a significant predictive factor for olfactory symptom improvement following sinus surgery for conductive disorders. To reveal the functional significance of IVO measures, we compared synaptopHluorin signals from OSN axon terminals in mice following intravenous odor injection under three pathological conditions: increased mucus, blocked orthonasal pathways (conductive disorders), and reduced number of OSNs (neural disorder). We found that onset latency of the response was prolonged only in mice with decreased OSN number. Furthermore, increased onset latency was correlated with decreased mature OSN numbers. These results indicated that latency of odor recognition onset in the IVO test is helpful for detecting neural olfactory disorders and that the magnitude of onset latency prolongation represents the extent of OSN injury.

## Results

### Onset latency of odor detection response using a pre-operative IVO test in patients having chronic rhinosinusitis with olfactory disorder correlates with post-operative olfactory symptoms

Chronic rhinosinusitis (CRS) is a major cause of olfactory disorders^30^,^31^,^32^. The etiologies of this disease are most likely conductive disorders and direct injury to OSNs. For treatment, endoscopic sinus surgery (ESS) is performed to improve airflow within the nasal cavity and ventilation among the sinus cavities^33^. This surgical technique can only improve loss of olfaction due to conductive disorders. Accordingly, CRS patients with accompanying direct olfactory epithelium (OE) injury could be expected to show worse olfactory outcome following ESS compared with CRS patients with accompanying conductive disorder only. To reveal predictive factors for the outcome in CRS patients with olfactory disorder, we retrospectively analyzed 482 patients with CRS who underwent ESS. The following factors were determined pre-operatively: age, sex, primary or revision surgery, presence/absence of polyps, Likert scale scores for nasal discharge and nasal obstruction, relative onset latency and duration of odor detection response in the IVO test, detection and recognition score in the T & T olfactory test, Lund-Mackay computed tomography (CT) score, IgE (IU/mL), percentage and number of eosinophils, presence/absence of allergic rhinitis, asthma, and diabetes mellitus, current smoking status, and Brinkman smoking index. The post-operative outcome at 6 months following ESS was evaluated using a Likert scale score of nasal symptoms (olfactory symptoms, nasal discharge, and nasal obstruction).

Of 482 patients screened, 99 patients without olfactory symptoms before ESS were excluded. Of the remaining 383, 101 were excluded because of absence of pre-operative evaluations (T & T test, 20 cases; IVO test, two cases; no response in IVO test, 67 cases; IgE, 12 cases). Of the remaining 282 patients, 29 were excluded for failure to evaluate post-operative Likert scale scores. Of 253 patients analyzed, pre-operative Likert scale scores (nasal discharge and obstruction) were significantly improved after ESS (nasal discharge, Supplementary Fig. 1a; nasal obstruction, Supplementary Fig. 1b; Mann-Whitney *U-*test, p < 0.001), indicating that ESS contributed to amelioration of conductive disorders. Table 1 shows univariate and multivariate tests of pre-operative factors associated with post-operative olfactory outcome.

In the univariate analysis, only relative onset latency was predictive of post-operative olfactory outcome (Table 1). Because other pre-operative factors (T & T detection, T & T recognition, and Brinkman index) also had relatively small p-values (p ≤ 0.2), multivariate analyses were conducted. Again, relative onset latency was the only factor predictive of post-operative olfactory outcome (Table 1). The optimum cut off value calculated from the area under the receiver operating characteristic curve (AUC) to determine appropriate thresholds for quantitative parameters (AUC, 0.65; 95% confidence interval, 0.58–0.72) was 1.6, which was taken as the relative onset latency (Supplementary Fig. 2a, odds ratio, 2.7; sensitivity, 64%; specificity, 60%), and the curative ratio following ESS in patients with a preoperative onset latency of less than 1.6 was better than that in patients with a preoperative onset latency of more than 1.7 (Supplementary Fig. 2b, Mann-Whitney *U-*test, p < 0.001). These results indicate that humans with prolonged onset latency in a pre-operative IVO test showed the worst outcome of olfactory symptoms following ESS, raising the possibility that extent of onset latency of the odor detection response in pre-operative IVO tests in patients with CRS would reflect involvement of an OSN disorder due to OE injury, while normal onset latency would reflect entirely conductive disorders.

### Intravenous administration of PST increases spH signals from OSNs

**Figure 2:**
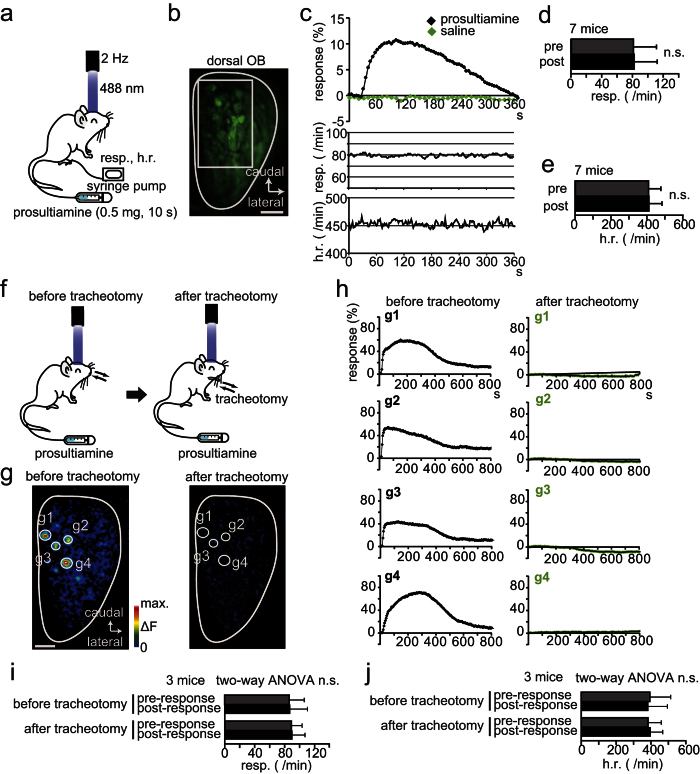
Intravenous injection of prosultiamine increases synaptopHluorin (spH) signals *via* the retronasal pathway. (**a**) Schematic of the experimental design. Prosultiamine (PST) was administered via the tail vein using a syringe pump. Respiration (resp.) and heart rate (h.r.) were monitored during experiments together with spH fluorescence (laser excitation 488 nm, emission 525/50 nm). (**b**) Confocal image of spH signals before PST administration. White rectangle shows a region of interest (ROI) for which responses are shown in (**c**). Scale bar, 500 μm. (**c**) PST-induced spH responses (% response, ∆F/F_0_) monitored together with resp. and h.r. Response curve following stimulation with PST (black diamonds) or saline (green diamonds). Each point (diamond) is the averaged response over 5 s (one bin). (**d,e**) Comparison of average resp. (**d**) and h.r. (**e**) at 30 s pre- and post-PST injection. (**f**) Schematic of the tracheotomy. PST was administered before tracheotomy (left) and again after tracheotomy (right). (**g**) Images of the olfactory bulb (OB) dorsal surface before (left) and after tracheotomy (right). Each circle indicates an individual responsive glomerulus (g1–g4). Scale bar, 500 μm. (**h**) PST-induced spH signals from individual glomerulus (g1–g4) before and after tracheotomy. SpH signals were detected from glomeruli g1–g4 before the tracheotomy, whereas responses were not observed from the same glomeruli after tracheotomy. (**i,j**) Comparison of respiration (resp., **i**) and heart rate (h.r., **j**) pre- and post-prosultiamine injection before and after tracheotomy (each value is the average of a 30 s epoch). Statistical comparisons were done using the Mann-Whitney *U*-test (**d**,**e**) and two-way ANOVA (**i**,**j**). n.s., no significant difference. All values are mean ± SD.

Odor information detected by OSNs is transmitted via their axons to the olfactory bulb (OB) and then via the axons of OB neurons to the olfactory cortex to generate the sensation of smell^34^,^35^,^36^,^37^. As a first step to reveal functional significance of IVO measures, we examined whether IVO measures can detect neural disorders by comparing synaptopHluorin signals from OSN axon terminals in adult mice. PST was administered through the tail vein using a syringe pump (Fig. 2a), and synaptic activity of OSN axon terminals at the dorsal surface of the OB of olfactory marker protein (OMP)-spH mice was measured using spH fluorometry. Respiration and heart rate were also monitored (Fig. 2a,b). In OMP-spH mice, odor-induced activation of OSNs caused increased spH signals of OSN axon terminals in the glomeruli of the OB^38^. After intravenous administration of PST, spH signals from OSN axon terminals started to increase at a latency of approximately 30 s, whereas saline administration did not induce clear spH signals (Fig. 2c). PST-induced responses might have been due to changes in respiration and heart rate, because OSN activity depends on pulmonary exhalation of the odorant. To rule out this possibility, we compared heart and respiration rates before and after PST injection (Fig. 2c–e). No significant differences were found (respiration rate, p = 0.9, Fig. 2d; heart rate, p = 0.89, Fig. 2e; Mann-Whitney *U*-test), indicating that OSN axon responses were directly induced by intravenous PST, not by associated changes in heart rate or respiration.

Next, we examined whether OSN axon terminal responses at the OB are induced by odorants widely released from blood vessels in the respiratory tract and carried to OSNs via the retronasal pathway or induced by an odorant leak from blood vessels within the nasal mucosa. As tracheotomy creates an airway that allows mice to breathe without use of the nose or mouth, if neural responses to intravenously administered PST are induced via the retronasal pathway, they should be completely abolished following a tracheotomy (Fig. 2f). Before tracheotomy, we observed clear neural responses in five mice following PST administration (Fig. 2g,h, g1–g4, left). After tracheotomy, however, neural responses to PST administration were absent (Fig. 2g,h, g1–g4, right), without alterations in heart rate or respiration (respiration: effect of tracheotomy, F_(1,297)_ = 1.71, p = 0.19; differences between pre- and post-response, F_(1, 297)_ = 0.5, p = 0.48, Fig. 2i; heart rate: effect of tracheotomy, F_(1,297)_ = 0.03, p = 0.86, differences between pre- and post-response, F_(1, 297)_ = 0.2, p = 0.66, Fig. 2j; two-way ANOVA). These results suggest intravenous PST odorants are released in the respiratory tract and carried to the olfactory epithelium via the retronasal pathway to activate OSNs.

**Figure 3:**
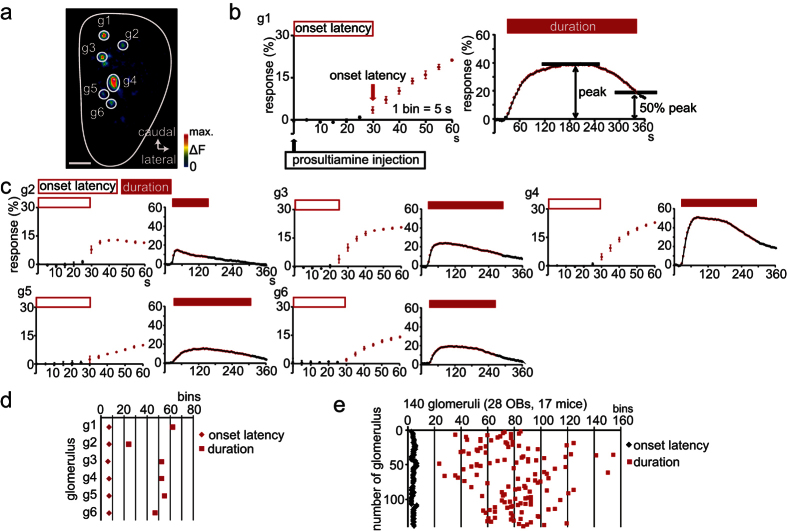
Individual glomeruli show distinct neural response patterns but similar onset times in response to prosultiamine (PST) stimulation. (**a**) Image of the olfactory bulb (OB) dorsal surface following PST administration. Glomeruli g1–g6 are responsive. Scale bar, 500 μm. (**b**) PST-induced spH signals from glomerulus g1. The onset latency was determined as the first 5 s bin with significantly increased ∆F/F_0_ compared with that of the bin immediately before PST administration (left, magenta arrow, onset time). The duration was determined by the interval from the onset time to 50% decay from peak (right). (**c**) PST-induced synaptopHluorin signals from individual glomeruli (g2 to g6; magenta open rectangles, onset time; magenta filled rectangles, duration). (**d**) Summary of onset latency and duration for each glomerulus shown in (**a**) (g1–g6). Diamond, onset time; square, duration. (**e**) Summary of onset latency and duration for 140 glomeruli (28 olfactory bulbs, 17 mice). Diamond, onset latency; square, duration.

Axons of OSNs expressing the same odorant receptor converge onto one or a few specific glomeruli, and each glomerulus represents a single odorant receptor^36^,^39^. Because different glomeruli represent different odorant receptors, individual glomeruli may show distinct response patterns to the same odorant. To characterize OSN axon activity patterns in individual glomeruli on the dorsal surface of the OB during PST stimulation (Fig. 3a), we measured onset latency, peak amplitude, and duration of the OSN axon response. We defined onset latency as the interval from the time of PST administration to the time of the first statistically significant increase in the OSN axon response (Fig. 3b, left, g1 shown in a). Response duration was defined as time from the response onset to 50% decay of the peak response, because it was occasionally difficult to determine an exact time of response termination (Fig. 3b, right; see methods). Individual glomeruli within the OB showed distinct response patterns to PST administration with highly variable response durations (g2–g6 in Fig. 3c,d). In contrast to these variable durations, we observed little variation in onset latency of OSN axon responses among these glomeruli (g1–g6 in Fig. 3c,d; Supplementary Movie 1). In addition, onset latency was relatively constant across OSN axon responses obtained from different mice (Fig. 3e; onset latency, 4.4 ± 1.0 bins; duration, 80.0 ± 24.3 bins; mean ± SD; 140 glomeruli from 17 mice).

### Increased mucus production shortens response duration but does not affect onset latency

**Figure 4:**
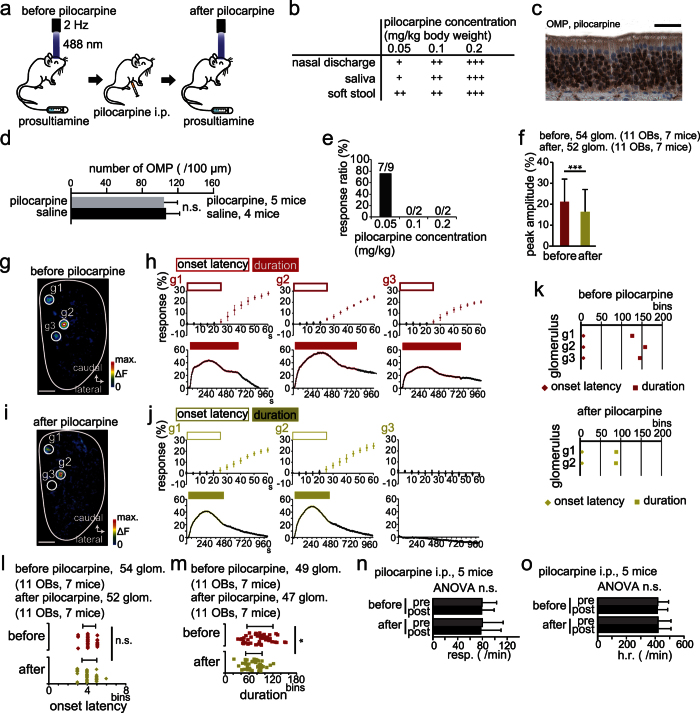
Increased mucus secretion induces shorter duration responses but does not affect onset time. (**a**) Experimental protocol for pilocarpine injection. (**b**) Physical responses (nasal discharge, saliva, and soft stool) to different pilocarpine concentrations (0.2, 0.1, and 0.05 mg/kg). +++, prominent; ++, intermediate; +, mild. (**c**) A representative photomicrograph of the OE stained for olfactory marker protein (OMP) in a pilocarpine-treated mouse. Scale bar, 20 μm. (**d**) Comparison of the number of OMP-positive cells between pilocarpine- and saline-injected mice. (**e**) Response ratio (magnitude after vs. before pilocarpine administration) at each concentration. Clear neural responses are detected only at 0.05 mg/kg (in seven of nine mice), whereas no responses are detected after the administration of high pilocarpine concentrations (0.2 and 0.1 mg/kg, two mice per dose group). (**f**) Comparison of peak amplitude between before and after pilocarpine administration. (**g**) Image of the OB dorsal surface before pilocarpine administration. g1–g3 are individual glomeruli. Scale bar, 500 μm. (**h**) PST-induced spH signals from individual glomeruli (g1–g3) before pilocarpine administration. Magenta open rectangle, onset latency; magenta filled rectangle, duration. (**i**) Image of the OB dorsal surface after pilocarpine administration. g1–g3 as in (**e**). Scale bar, 500 μm. (**j**) PST-induced spH signals from individual glomeruli (g1–g3) after pilocarpine administration. Yellow open rectangle, onset latency; yellow filled rectangle, duration. (**k**) Summary of onset latency and duration for each glomerulus shown in (**g,i**). Diamond, onset latency; square, duration. (**l,m**) Comparison of onset latency (**l**) and duration (**m**) before and after pilocarpine administration. OB, olfactory bulb. (**n,o**) Comparison of respiration (resp., **n**) and heart rate (h.r., **o**) before and after pilocarpine administration. Each recording includes the period of pre- (pre) and post-response (post). Statistical comparisons were done using Mann-Whitney *U*-test (**d,f,l**,**m**) and two-way ANOVA (**n**,**o**). n.s., no significant difference. *p < 0.05. All values are mean ± SD.

Increased mucus in the nasal cavity is common to many acute and chronic olfactory disorders, such as the common cold, rhinitis, and allergies. This type of olfactory disorder is classified as conductive because excess mucus could impede binding of odorants to target receptors on OSNs. To examine whether increased mucus affects onset latency and duration of OSN responses elicited by intravenous PST injection, we measured parameters of responses before and after induction of increased mucus production using the muscarinic agonist pilocarpine (Fig. 4a).

As a control, we first injected seven mice with saline instead of pilocarpine (Supplementary Fig. 3a). The first administration of PST induced clear OSN axon responses in all six recorded glomeruli (g1–g6 in Supplementary Fig. 3b,c), with stable onset times and relatively variable response durations (Supplementary Fig. 3c,d). After intravenous saline injection, the second administration of PST also induced clear OSN axon responses (Supplementary Fig. 3b,c), with stable onset latency and variable duration (Supplementary Fig. 3c,d). As expected, we observed no significant difference in magnitude, onset latency, and duration of the response before and after saline administration, and no significant alterations in heart rate or respiration (response magnitude: p = 0.75, Supplementary Fig. 3e; onset latency: p = 0.71, Supplementary Fig. 3f; duration: p = 0.3, Supplementary Fig. 3g; Mann-Whitney *U-*test; respiration, effect of saline administration, F_(1, 357)_ = 0.52, p = 0.47, differences between pre- and post-response, F_(1, 357)_ = 0.57, p = 0.45, Supplementary Fig. 3h; heart rate, effect of saline administration, F_(1, 357)_ = 0.64, p = 0.43, differences between pre- and post-response, F_(1, 357)_ = 0.17, p = 0.69, Supplementary Fig. 3i; two-way ANOVA).

Administration of pilocarpine induced concentration-dependent increases in saliva and mucus secretion, as well as soft stool (Fig. 4b and Supplementary Fig. 4), but without prominent injury to the olfactory epithelium [OMP-positive cells, pilocarpine (5 mice) vs. saline (4 mice), p = 0.47, Fig. 4c,d; Mann-Whitney *U-*test]. Pilocarpine at 0.2 and 0.1 mg/kg induced prominent nasal discharge (Fig. 4b) and completely blocked the OSN axon response following PST administration (Fig. 4e). At 0.05 mg/kg pilocarpine, seven of nine mice showed clear OSN axon responses to PST injection (Fig. 4e), although responses were lower in magnitude than pre-pilocarpine responses (Fig. 4f; Mann-Whitney *U*-test, p < 0.05). These results indicated that the OSN responses to PST injections under conditions of increased mucus production are weaker because mucus may prevent odorants from reaching odorant receptors on OSNs, as in a conductive disorder.

Representative OSN axon responses in glomeruli before and after pilocarpine administration (0.05 mg/kg body weight) are shown in Fig. 4g,i. Before pilocarpine administration, PST-evoked activity was detected in three glomeruli (g1–g3 in Fig. 4g,h,k) with very similar onset latencies but different durations. After pilocarpine administration, the OSN axon response was still detected in two of these glomeruli with similar onset latencies but, again, different durations (g1–g2 in Fig. 4i–k). On average, onset latency of the OSN axon response after pilocarpine administration did not differ significantly from that before pilocarpine administration (Fig. 4l; Mann-Whitney *U-*test, p = 0.78), whereas the mean duration was significantly shorter (Fig. 4m; Mann-Whitney *U-*test, p < 0.05).

The effects of pilocarpine on the autonomic nervous system, such as increased heart rate or respiration, might alter the latency and duration of the OSN axon response. To examine this possibility, we compared heart and respiration rates between responses before and after pilocarpine administration (Fig. 4n,o). Although we cannot rule out the possibility that a high dose of pilocarpine could induce cardiovascular changes with muscarinic action in addition to increased salivary secretion, the dosage used in this experiment would not be enough to induce significant changes in respiration and heart rate between pre- and post-response (respiration: effect of pilocarpine administration, F_(1, 297)_ = 1.45, p = 0.23, differences between pre- and post-response, F_(1, 297)_ = 0.03, p = 0.87, Fig. 4n; heart rate: effect of pilocarpine administration, F_(1, 297)_ = 0.16, p = 0.69, differences between pre- and post-response, F_(1, 297)_ = 0.03, p = 0.87, Fig. 4o; two-way ANOVA). Taken together, these results indicate that increased mucus reduces response duration but does not affect response onset latency.

### Orthonasal airflow blockage shortens response duration but does not affect onset latency

Nasal airflow is chronically blocked under pathological conditions such as nasal congestion, swelling, and polyps. Pathological nasal blockage frequently occurs at the relatively rostral part of the nasal cavity, and human OSNs are more numerous in the dorsoposterior regions of the nasal septum and superior turbinate than in the anterior portions of the septum or the middle turbinate^40^. These clinical conditions, therefore, usually obliterate the sense of smell in response to orthonasal stimulation (e.g., T & T olfactometry), but diminish sensation in response to retronasal stimulation (e.g., IVO test)^41^.

**Figure 5:**
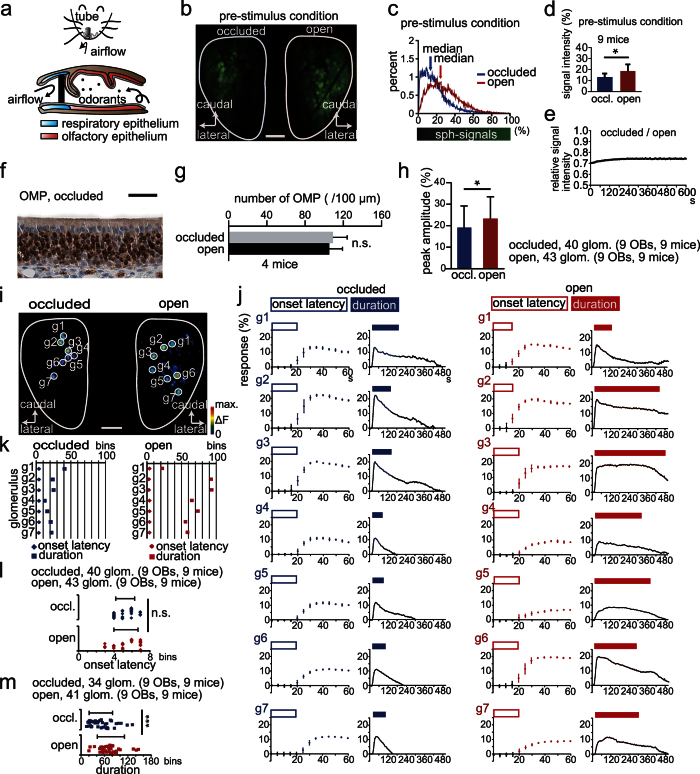
Orthonasal airflow blockage reduces response duration but has no effect on onset latency. (**a**) Schematic of orthonasal airflow blockade. Upper, a silicon tube is inserted into the rostral part of the unilateral nostril; Lower, silicon-tube insertion blocks orthonasal airflow. (**b**) Image of the OB dorsal surface before prosultiamine (PST) administration. Scale bar, 500 μm. (**c**) Intensity histograms of spH signals from the dorsal surface of the OB on the occluded and open sides. Blue arrow shows median signal intensity of the occluded side, magenta arrow shows that of the open side. (**d**) Comparison of signal intensities between occluded (blue) and open sides (magenta). occl., occluded side. (**e**) A representative trace of the relative spH-signal intensity from the dorsal surface of the OB on the occluded side. The trace is represented as intensity of the occluded side compared with the open side. Note that nostril occlusion without PST administration did not induce significant signal-changes over a period of 600 s. (**f**) A representative photomicrograph of the OE stained with anti-OMP on the occluded side. Scale bar, 20 μm. (**g**) Comparison of the number of OMP-positive cells between occluded and open sides. (**h**) Comparison of peak PST-induced spH signals between occluded (blue) and open sides (magenta). (**i**) Images of the OB dorsal surface on the occluded (left) and open side (right). g1–g7 are individual glomeruli. Scale bar, 500 μm. (**j**) PST-induced spH signals from individual glomeruli on the occluded (left, g1–g7) and open sides (right, g1–g7). Open rectangle, onset latency; filled rectangle, duration. (**k**) Summary of onset latency and duration for each glomerulus shown in (**h**,**i**). Diamond, onset time; square, duration. (**l,m**) Comparison of onset latency (**k**) and duration (**l**) between occluded and open sides. There is no significant difference in the onset time between occluded and open sides, whereas the duration is significantly shorter on the occluded side. Mann-Whitney *U*-test (**d,f,g,k**,**l**). n.s., no significant difference, *p < 0.05, ***p < 0.001. All values are mean ± SD.

We previously developed a mouse model of unilateral nasal obstruction by inserting a silicon tube that completely abolishes orthonasal airflow and selectively disrupts orthonasally-induced OSN activity on the occluded side (Fig. 5a)^42^. Using the inserted silicone tube method, we examined how complete blockage of unilateral orthonasal airflow affects OSN axon responses to intravenously administered PST. Ongoing spH signals were detected on the occluded side even without PST administration due to room air odorants or periodic mechanical stimulation of the OSNs (Fig. 5b)^43^. However, these signals were significantly weaker than those on the contralateral, open side (Fig. 5c,d; Mann-Whitney *U-*test, p < 0.05). Furthermore, nostril occlusion without PST administration did not induce significant signal-changes on the occluded side over 600 s (three mice, Fig. 5e), which indicates that room air odorants and periodic mechanical stimulation of the OSNs under occluded conditions do not affect spH-signals. Because the number of cells expressing OMP, a marker of mature OSNs, on the occluded side did not change compared with that on the open side (Fig. 5f,g; 4 mice, Mann-Whitney *U-*test, p = 0.34), and this decrease in the OSN axon activity is consistent with conductive disorders.

We next compared PST-evoked OSN axon responses in glomeruli between open and occluded sides (Fig. 5h). Mean peak amplitude of PST-evoked responses on the occluded side was significantly lower than that on the open side (Fig. 5h; Mann-Whitney *U-*test, p < 0.05). Representative images of PST-induced activated glomeruli (g1–g7) and time course of OSN axon responses on the occluded and open sides (g1–g7) are shown in Fig. 5i,j. Despite weaker responses, onset latency did not differ among activated glomeruli on the occluded side compared with those on the open side (Fig. 5i–k and Supplementary Movie 2). In nine such mice, onset latency did not significantly differ between the occluded and open sides (Fig. 5l; Mann-Whitney *U-*test, p = 0.33), whereas duration on the occluded side was significantly shorter compared with that on the open side (Fig. 5m; Mann-Whitney *U-*test, p < 0.001).

Airflow through the right and left nasal passages is usually asymmetrical, as unilateral partial obstruction regularly occurs even in normal physiological conditions^44^. To examine whether PST-evoked OSN axon responses in the glomeruli were similar on the right and left sides under normal physiological conditions, we compared OSN axon responses between the right and left OBs in control mice (Supplementary Fig. 5a–c). Peak amplitude, onset latency, and duration of OSN axon responses did not differ significantly between the right and left sides (peak amplitude: p = 0.24, Supplementary Fig. 5d; onset latency: p = 0.61, Supplementary Fig. 5e; duration: p = 0.51, Supplementary Fig. 5f; Mann-Whitney *U-*test). Consistent with results from mice having excess mucus secretion, orthonasal airflow blockage induced responses with shorter duration but did not alter response onset latency. Thus, two distinct conduction disorders did not alter onset latency of the OSN axon response to PST injection.

### The number of mature OSNs regulates onset latency of the PST-evoked response

**Figure 6:**
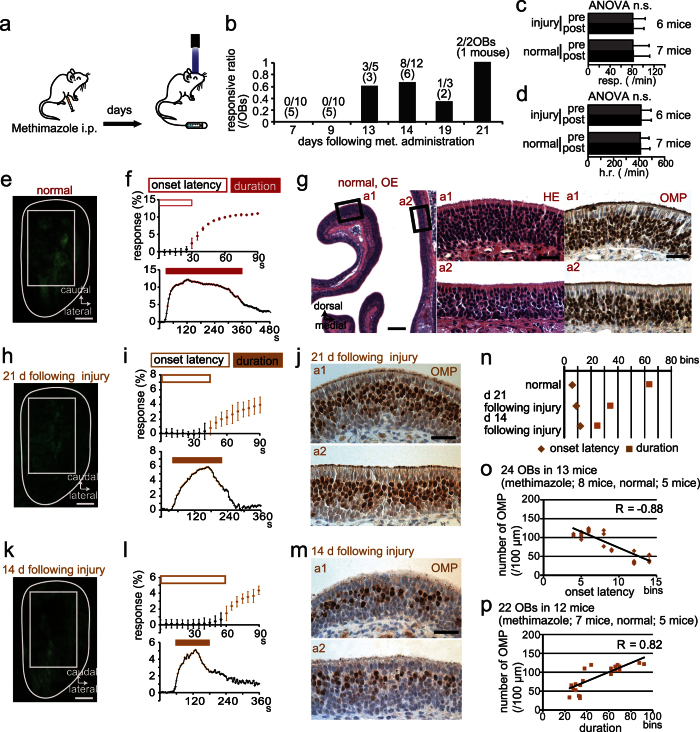
Number of mature olfactory sensory neurons correlates with changes in onset latency and response duration. (**a**) Schematic of the experimental protocol for methimazole (met.)-induced injury of olfactory sensory neurons (OSNs). (**b**) Summary of the change in response ratio following injury. Shown are responsive olfactory bulbs per the total number of olfactory bulbs examined. Numbers of mice are in parentheses. (**c,d**) Comparison of respiration (resp., **c**) and heart rate (h.r., **d**) pre- and post-prosultiamine injection between injury and normal mice (each value is the average of a 30 s epoch). (**e,f**) Image of the OB dorsal surface in a control mouse (**e**) and corresponding prosultiamine (PST)-evoked synaptopHluorin (spH) responses (**f**). White rectangle shows the ROI. Scale bar, 500 μm. Open rectangle, onset latency; filled rectangle, duration. (**g**) Photomicrograph of a representative coronal section of the olfactory epithelium (OE). Analyzed areas (a1, concha bullosa; a2, nasal septum) are shown in the left images. Higher magnification images of the areas enclosed by black squares (a1 and a2) stained with hematoxylin and eosin (HE, middle) or anti-olfactory marker protein (OMP, right) are shown. Scale bars, 100 μm at lower magnification, 20 μm at higher magnification. (**h,i**) Image of the OB dorsal surface at 21 days (d 21) after injury (**h**) and corresponding PST-evoked spH responses (**i**). Scale bar, 500 μm. (**j**) Anti-OMP-stained sections at 21 days after injury (a1, concha bullosa; a2, nasal septum). Scale bar, 20 μm. (**k, l**) Image of the OB dorsal surface 14 days (d 14) after injury (**k**) and corresponding PST-evoked spH responses (**l**). Scale bar, 500 μm. (**m**) Anti-OMP-stained sections 14 days after injury (a1, concha bullosa; a2, nasal septum). Scale bar, 20 μm.(**n**) Summary of onset latency and duration on different days following injury. Diamond, onset latency; square, duration. (**o,p**) Correlation between number of OMP-positive cells and onset latency (**o**) and duration (**p**). n.s., no significant difference. All values are mean ± SD.

The OE is directly exposed to neurotoxic compounds in ambient air, making peripheral olfactory neurons highly vulnerable to injury. Thus, OSN numbers frequently diminish. The olfactotoxic drug methimazole disrupts virtually all OSNs across the OE by activating the apoptotic cascade^42^,^45^. To compensate for OSN loss, surviving basal progenitor cells generate new OSNs, resulting in gradual return of OE function, usually by 28 days after methimazole administration^42^. It is thus possible to roughly predict state of the OE recovery from the number of days following methimazole-induced injury (Fig. 6a). We measured PST-induced spH signals of OSN axons on days 7, 9, 13, 14, 19, and 21 following methimazole-induced injury (Fig. 6b).

Newly generated OSNs begin to become mature sensory neurons between days 7–14 after the injury^42^. While it is possible that even a quite small number of functionally mature OSNs could induce spH-changes, we could not detect significant PST-induced spH signals of OSN axons until day 13 (Fig. 6b). Although responsive ratios at 19 and 21 days following methimazole administration were obtained from a small number of mice (Fig. 6b), these results indicate that PST-induced responses require a minimum number of functionally mature OSNs. We thus examined how PST-induced OSN axon responses changed beyond 13 days following injury. There were no significant differences in respiration or heart rate pre- and post-PST injection between mice beyond 13 days after injury and non-injured, normal mice, indicating methimazole injection induced OE injury without influencing the autonomic nervous system (respiration: injury vs. normal, F_(1, 387)_ = 0.49, p = 0.49, differences between pre- and post-response, F_(1, 387)_ = 0.1, p = 0.75, Fig. 6c; heart rate: injury vs. normal, F_(1, 387)_ = 0.07, p = 0.8, differences between pre- and post-response, F_(1, 387)_ = 0.08, p = 0.77, Fig. 6d; two-way ANOVA). After recording OSN axon responses, we examined immunohistological changes of the OE structure in 13 mice. For spH signal recordings, a relatively broad area of the dorsal OB surface was selected as the region of interest (Fig. 6e,h,k) because we could not clearly identify individual activated glomeruli in methimazole-injected mice, presumably due to incomplete regeneration of functional OSN axons and concomitantly weaker OSN axon responses^42^.

Representative images of the OB dorsal surface and PST-induced OSN axon responses in a control mouse are shown in Fig. 6e,f. In the control mouse, the PST-evoked OSN axon response started at a latency of 30 s and lasted for 320 s. Coronal sections of OE (Fig. 6g) revealed a substantial number of OMP-positive mature OSNs (Fig. 6g; mean: 115 per 100 μm).

At 21 days after methimazole-induced injury (Fig. 6h), we observed a spH signal at the dorsal surface of the OB, although the signal was still relatively weak (Fig. 6i). Onset latency (~45 s) of the OSN axon response was longer, and duration (~170 s) was shorter than in control mice (Fig. 6i). At post-injury day 21, only about half of the OSNs were OMP-positive (mature), and the total number of OSNs was reduced compared with that of controls (Fig. 6j; mean number of OMP-positive cells, 65 per 100 μm). Thus, lower numbers of mature OSNs resulted in not only shortened response duration but also prolonged onset latency.

At 14 days after injury (Fig. 6k), we observed similar results, with prolonged onset latency and shorter duration (onset latency, ~60 s; duration, ~120 s; Fig. 6l). Moreover, only about one quarter of the OSNs was functionally mature (Fig. 6m; mean number of OMP-positive cells, 33 per 100 μm). In summary, comparison of onset latency and duration of PST-evoked OSN axon responses between control and methimazole-treated mice (at both 14 and 21 days following injury) revealed that extent of the onset latency prolongation and amount of the response duration shortening were associated with the number of days following injury (Fig. 6n). This in turn suggests that the magnitudes of onset latency prolongation and the response duration shortening increase as the number of functional OSNs was reduced. In support of this, there were significant correlations between decrease in the number of mature OSNs and both onset latency and duration of PST-evoked OSN axon responses (Fig. 6o, onset, R = −0.88; Fig. 6p, duration, R = 0.82). Because onset latency was not influenced by the conduction block caused by increased mucus or orthonasal airflow blockage (Figs 4 and 5), these results indicate that onset latency of odor detection response following intravenous injection of PST is a useful biomarker to detect neural disorder, and that magnitude of onset latency prolongation may predict magnitude of OSN loss.

## Discussion

**Figure 7:**
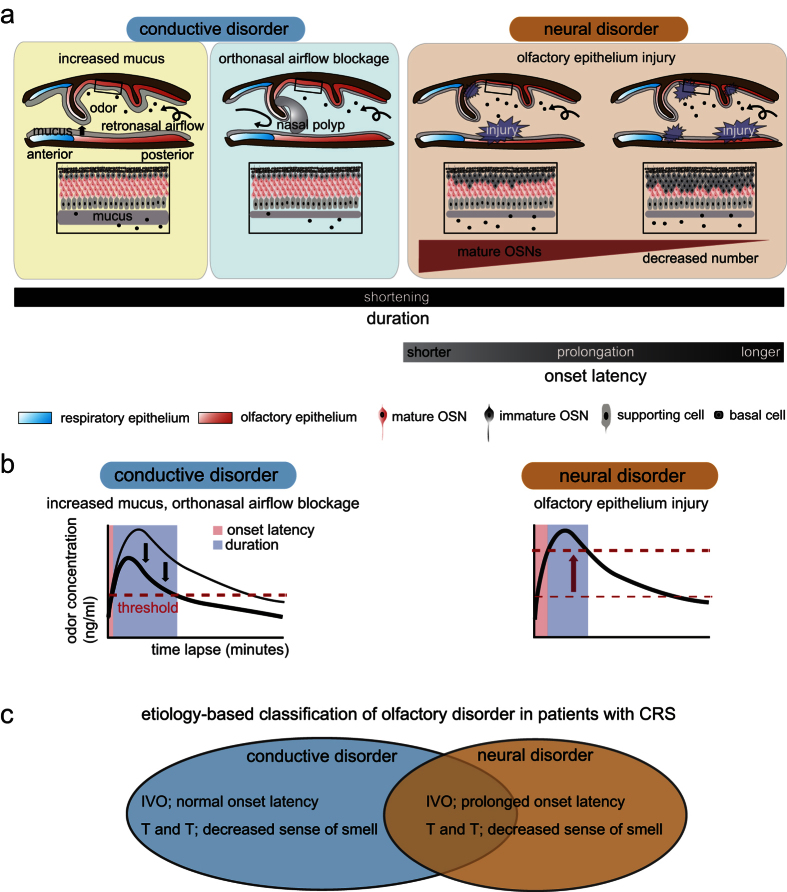
Schematic diagrams of hypothetical mechanisms for changes in onset latency and duration under different pathological conditions. (**a**) Sagittal views of the nasal cavity are shown for different pathological conditions (left, anterior; right, posterior). Higher magnification of the olfactory epithelium (OE) area indicated by the square in the upper schematic is shown in the lower panel. Conductive disorders result from increased mucus and orthonasal airflow blockage. The duration is shortened by both conductive and neural disorders, whereas onset latency is changed (prolonged) only by neural disorders. Additionally, the extent of the decrease in mature OSN number correlates with the extent of onset latency prolongation. (**b**) Hypothetical odorant concentration curves with time under different pathological conditions. With increased mucus or orthonasal airflow blockage (left), the entire concentration curve is shifted downward. These situations can induce prominent shortening of neural response duration but do not change the onset latency. In olfactory epithelium injury (right), the response threshold of individual glomeruli can be shifted markedly higher, resulting in a prominent shortening of response duration and the prolongation of onset latency. Dashed line, glomerular activation threshold. (**c**) Etiology-based classification of olfactory disorder in patients with CRS. Cases with normal onset latency in the IVO test but decreased sense of smell in T and T test would correspond to the “type without neural disorder (or conductive disorder only).” Conversely, cases with prolonged onset latency in the IVO test, and decreased sense of smell in T and T test, would correspond to the “type with neural disorder.”

Schematics of the conductive and OSN disease models are shown in Fig. 7a. Conductive disorders due to increased mucus or orthonasal blockage reduced response duration but did not alter onset latency of the OSN axon response (Figs 4 and 5). In striking contrast to this result, a methimazole-induced reduction in the number of mature functional OSNs as a model of OSN disorders resulted in reduced response duration and clear prolongation of onset latency (Fig. 6). Furthermore, extent of onset latency prolongation correlated significantly with decrease in the number of mature OSNs (Fig. 6). Here, it is interesting to consider the idea that evoked potentials and latency of sensory responses could be used to determine neural disorders in the somatosensory system. For instance, it is possible that a neural disorder in the spinal cord could affect the amplitude of somatosensory evoked potentials in the brain, despite the large distance between cortical neurons and interneurons in the lumbar spinal cord^46^. Although it is unclear how initiation and termination of OSN responses correlate with the subjective sensation of smell, and whether neural response changes in imaging studies using spH fluorometry correlate with those in electrophysiological methods, we propose that onset latency of the odor detection response in IVO tests helps to detect OSN disorders and indicate extent of OSN injury.

Previous reports indicate that when PST is intravenously administered to humans, concentration of mercaptan (metabolites of PST) in expired air rapidly increases to a peak within approximately 60 s before gradually decreasing^22^,^29^. An ascending portion of the odorant concentration curve could regulate onset latency of OSN responses, whereas a descending portion could regulate termination time. We speculate that the time course of rapidly increasing and gradually decreasing odorant concentrations^22^,^29^ may explain changes in onset latency of OSN responses under various pathological conditions and the associated alterations in odorant detection onset and duration.

Figure 7b shows a hypothetical time course derived from previous reports^22^,^29^ of odorant concentration change in the OE during exhalation and possible changes in the time course under pathological conditions. For increased mucus secretion, the response magnitude was smaller than that in the control condition (normal mucus) (Fig. 4f). Under excessive mucus secretion conditions induced by high concentrations of pilocarpine, some mice shifted to mouth-breathing, and glomerular spH responses were not detected (Fig. 4e), presumably due to complete blockage of airflow through the orthonasal and retronasal pathways. Increased mucus somehow accompanies decreased ortho- or retronasal airflow (or both), and it is likely that increased mucus prevents odorants from accessing target odorant receptors. In either case, the reduced concentration of odorants at the OE was below the threshold for inducing detectable glomerular spH signals. Therefore, this condition may induce a downward shift in the odorant concentration curve (Fig. 7b, left). It should be noted that, despite decreases in the amplitude and duration of OSN axon response, all recorded glomerular spH responses showed relatively constant onset latency (Fig. 4l), presumably because of the presence of large numbers of mature functional OSNs.

Under conditions of orthonasal airflow blockage, spontaneous spH signals and PST-induced OSN axon responses were significantly reduced on the blocked side compared with those on the open side (Fig. 5d,g). This is presumably due to decreased odorant entry via the retronasal pathway. This condition may induce a downward shift in the odorant concentration curve on the occluded side, resulting in decreased amplitude and duration of the OSN responses (Fig. 7b, left). However, OSN axon responses showed relatively constant onset latency, again, presumably because of the presence of functional OSNs.

In the case of decreased numbers of mature OSNs, we speculate that higher odorant concentrations would be required to induce equivalent OSN axon responses in the glomeruli, because of the reduction of the number of responsive OSNs. This condition could thus induce a large upward shift in the response threshold, thereby prolonging the onset latency as well as reducing the response duration (Fig. 7b, right).

In this study, we established relatively simple disease models and examined influences on onset latency and duration of PST-evoked OSN axon responses in each model. However, sensory loss associated with CRS undoubtedly involves more complex heterogeneous inflammatory processes, including restricted mucociliary clearance^47^,^48^, a direct antipathogenic effect^48^,^49^, and abnormalities in the sinonasal epithelial cell barrier^48^,^50^. Typical CRS may thus arise from a variable combination of conductive disorders (e.g., increased mucus and complete airflow blockage) or conductive and neural disorders, which could alter onset latency as well as duration of PST-evoked responses. However, in clinical analyses of CRS patients, the onset latency of odor detection in the pre-operative IVO test was found to be a significant predictive factor for olfactory symptom improvement following sinus surgery for conductive disorder, consistent with our mouse experiments showing that only injury to OSNs (and not conduction block alone) could prolong the onset latency. We speculate that, even in cases of a combination of distinct conductive disorders or conductive and neural disorders, such conductive disorders will not contribute to significant onset latency changes of the spH response.

Olfactory disorders are classified as sinonasal^31^,^32^, post-viral upper respiratory tract infection (URTI)^51^, post-traumatic^52^,^53^, toxic^54^, and others^30^. Regardless of diagnosis, most cases are accompanied by direct injury to OSNs. Biopsies of human olfactory mucosa revealed squamous epithelium metaplasia, local pathological responses to various degrees of chronic inflammation in sinonasal disease^48^,^55^,^56^,^57^,^58^,^59^, reduced OSN numbers and occasional squamous epithelial metaplasia in URTI^57^, and disorganization and thickening of the OE in post-traumatic olfactory disorder^60^.

A reliable biomarker for the determination of OSN disorder or severity of OE injury could contribute to an etiology-based classification of patients with olfactory disorder. In clinical analyses of CRS patients with smell loss, the detection and threshold scores in the T and T test did not correlate with the olfactory outcome following sinus surgery for conductive disorder, whereas onset latency in the IVO test strongly correlated with the olfactory outcome. These findings may result from the differences in etiology intended by the individual olfactory tests. Cases with prolonged onset latency in the IVO test would correspond to the “type with neural disorder” (Fig. 7c, right). Conversely, cases with normal onset latency in the IVO test but decreased sense of smell in response to orthonasal stimulation would correspond to the “type without neural disorder (or conductive disorder only)” (Fig. 7c, left). We hypothesized that orthonasal stimulus tests (T and T test) could detect both conductive and neural disorders, analogous to air-conduction tests for the auditory system, whereas IVO retronasal stimulus tests could selectively detect neural disorders, analogous to bone-conduction tests for the auditory system. We can speculate that stronger OSN stimulation, and rapidly increasing concentration by an instantaneous and abundant release of intravenously administered odors into the nasal cavity compared with that in T and T tests could contribute to selective detection of etiologies.

Etiology-based classification, as opposed to diagnoses based on primary cause, could provide useful information for choosing optimal therapeutic strategies. For conductive disorders, in which there is substantial potential for improving olfactory symptoms, anti-inflammatory drugs, antibiotics, surgical procedures, or some combination could be applied according to the patient’s condition^33^,^61^,^62^,^63^. For neural disorders, neurotrophic factors and continuous odor stimulation could be applied to facilitate regeneration of OSNs^42^,^64^,^65^,^66^.

Another application of the onset latency results in the IVO test is forming a prognosis. In our analysis, post-operative olfactory symptoms in patients with CRS correlated with pre-operative IVO test onset latency. If the pre-operative onset latency is normal, olfactory symptoms would be expected to immediately improve following surgery. In other olfactory disorders, such as URTI or post-traumatic olfactory disorder, the prognosis may also be predicted by focusing on the extent of onset latency prolongation. We speculate that cases with severely prolonged onset latency might require a longer period for the improvement of olfactory symptoms.

The major disadvantages of the IVO test are that it requires patient collaboration and its impact is limited by the possible biasing of results through its subjective nature. However, as with orthonasal olfaction tests, the IVO test is simple, safe, and covered by health insurance in Japan. Compared with orthonasal olfaction tests, the retronasal IVO test is unique in that measuring onset latency can predict olfactory improvement following surgery and provide clues to disease etiology.

## Methods

### Participants and analyses performed in the clinical study

This study retrospectively analyzed 482 patients with CRS at Jikei University Hospital between April 2007 and March 2008. Diagnoses of sinus disease were based on patient history, clinical examination, nasal endoscopy, and CT of the sinuses according to the guidelines of the European Position Paper on Rhinosinusitis and Nasal Polyps^67^. All patients were unresponsive to previous conservative therapy and underwent ESS. Patients were excluded if they were treated with oral steroids or antimicrobial agents within 4 weeks before surgery, were 18 years of age or younger, or had unilateral olfactory diseases, fungal diseases, antrochoanal polyps, allergic fungal rhinosinusitis, or cysts of the paranasal sinuses. Patients with aspirin-exacerbated respiratory disease were also excluded because the pathophysiology differs from that of aspirin-tolerant asthma^68^. After ESS, a Celestamine combination tablet, which contains betamethasone (0.25 mg) and d-chlorpheniramine maleate (2 mg) (Merck & Co., Inc., Tokyo, Japan), mucolytics, and anti-allergic agents were orally administered daily for 2 weeks to all patients.

Pre-operative demographic information and medical histories were obtained from each patient, including age, sex, history of prior sinus surgery, allergic rhinitis, asthma, and smoking habits. Allergic rhinitis was confirmed by intradermal skin testing, and serum total IgE and specific IgEs for common allergens were measured using fluoroenzyme immunoassays. The CT findings were assessed according to the degree of total opacification (the Lund-MacKay method)^69^, which was calculated based on the sum of the scores of the five right and left sinuses and scored on a 5-point opacification scale as follows: 0 = 0%, 1 = 1–25%, 2 = 26–50%, 3 = 51–75%, 4 = 76–99%, and 5 = 100%. All patients included in the analysis were monitored for at least 6 months after ESS.

Two types of olfaction tests were performed in patients before the ESS: modified T & T olfactometer (detection and recognition thresholds) tests and IVO tests. The T & T test used the following three odorants^10^,^70^: (A) β-phenyl ethyl alcohol, which smells like a rose; (B) methyl cyclopentenolone, which smells like burning sugar; and (C) isovaleric acid, which smells like sweat (Takasago Industry, Tokyo, Japan). The range of concentrations for each odorant was over eight degrees of intensity (−2–5) except for odorant (B), which was over seven degrees (−2–4). These odorants were presented to the entrance of the participant’s nostrils and orthonasally stimulated the OSNs. The detection threshold was defined as the lowest concentration detectable by the subject, whereas the recognition threshold was defined as the lowest concentration at which the odor could be identified. Subsequently, the detection and recognition thresholds in three odorants were averaged, and the olfactory acuity was evaluated using the averaged values. The IVO test was performed pre-operatively using PST (Alinamin, Takeda Pharmaceutical Company Limited, Osaka, Japan), which smells like garlic or onion. A dose of 10 mg (2 mL) of PST was injected into an antecubital vein at a constant rate over 20 s. Onset was defined as the first report of a definite smell of garlic/onion rather than an undefined odor perception, because PST can also stimulate the trigeminal nerve. The duration of smell sensation from initiation to termination was also measured. Based on previous data in healthy groups, we regarded less than 10 s as normal onset time and more than 60 s as normal duration^22^,^28^,^71^. In this analysis, we used relative onset and duration from normal.

All patients were interviewed within 1 month before ESS and at 6 months after ESS to evaluate subjective nasal symptoms (nasal discharge, nasal obstruction, and decreased sense of smell). Nasal symptoms were assessed using a 7-point Likert scale of symptom severity (from 0 for no nasal discharge, no nasal obstruction, and normal sense of smell to 6 for prominent increase in nasal discharge, complete nasal obstruction, and anosmia)^72^. Logistic regression analyses were performed to identify factors predictive of outcome. The olfactory outcome in logistic regression analyses was coded as 0 or 1, where 1 indicated a Likert scale score of 1 to 6 following ESS, and 0 indicated a Likert scale score of 0 (normal sense of smell) following ESS. Predictive variables were as follows: age, sex, primary or revision surgery, presence/absence of polyps, Likert scale scores for nasal discharge and nasal obstruction, relative onset time and duration in the IVO test, detection and recognition score in the T & T olfactory test, Lund-Mackay CT score, IgE (IU/mL), percentage and number of eosinophils, presence/absence of allergic rhinitis, asthma, and diabetes mellitus, smoking status, and Brinkman smoking index. Associations between predictive variables and postoperative outcomes of olfactory symptoms were expressed as odds ratios (OR) and respective ninety-five percent confidence intervals (95% CI).

We defined p as the probability that the outcome was one. A p-value of <0.05 was considered statistically significant. The receiver operator characteristic (ROC) curve is a plot of sensitivity (true positive rate) and one minus specificity (true negative rate) for each possible threshold value of the onset latency. The area under the ROC curve (AUC), sensitivity, and specificity were used to evaluate the performance of the model. The curative ratio following ESS was calculated as the ratio of Likert scale scores of 0 (normal sense of smell) following ESS for each threshold value of the pre-operative onset latency.

### Animals

Adult heterozygous OMP-spH 10-week-old knock-in male mice (Jackson Laboratories, Bar Harbor, ME, USA) were anesthetized with Nembutal (1 g/kg, intraperitoneal)^38^ and 1–2.5% isoflurane (Supplementary Tables 1 and 2). The animals were kept on a heating pad throughout the procedures, and both respiration and heart rates were continuously monitored (Mouse STAT; Kent Scientific, Torrington, CT, USA). The bone covering the dorsal OB was thinned and perforated using a dental drill. A catheter was inserted into the lateral tail vein using a 30-gauge needle connected to a non-toxic medical-grade polyethylene tube (SP10; Natsume Seisakusho Co., Ltd., Tokyo, Japan). PST (0.5 mg, Alinamin; Takeda Pharmaceutical Co., Ltd., Osaka, Japan) was administered via the tail vein catheter over 10 s using a syringe pump (KDS Syringe Pump; KD Scientific Inc., Holliston, MA, USA).

### Tracheotomy

The mice were laid supine on a table and anesthetized with 1–2.5% isoflurane. The trachea was exposed and then completely amputated under the cricoid cartilage. The cannula of a 20 gauge needle (Terumo Co., Ltd., Tokyo, Japan) was inserted into the remaining trachea, connected with the lung, and fixed with a thread to prevent the detection of odors leaking from the gap between the tube and trachea. Silicon tubes were inserted into both right and left nostrils to block odors leaking from the trachea. The mouse could breathe freely through this tube.

### Pilocarpine administration

Between the first and second recordings, pilocarpine (0.2, 0.1, or 0.05 mg/kg) was intraperitoneally injected to establish a model of conductive disorder caused by increased mucous secretion. Saliva and nasal discharge increased and watery stool was exhausted about 5 min after the injection. The second recording was performed 20 min after the pilocarpine injection. Owing to ongoing secretion from the nasal glands and continuous mucociliary clearance from the nose to the pharynx, it is difficult to subjectively evaluate nasal discharge, saliva secretion, and soft stool. These were thus evaluated using objective scores (+, secretions barely detectable by the unaided eye; ++, intermediate level of secretions; +++, prominent increase in secretions) (Fig. 4b and Supplementary Fig. 5). Mice were injected with PST twice, once before and once after pilocarpine or saline administration.

### Nostril occlusion

Mice were anesthetized with Nembutal, and a custom-made 10 mm silicon tube filled with glue was inserted into one nostril using a previously reported procedure^42^,^73^. Mice with unilateral nostril occlusion were perfused for histochemical analysis after spH recordings (4 mice, Fig. 5g, and Supplementary Table 2).

### Methimazole administration

To model a neural disorder, mice were intraperitoneally injected with methimazole (75 mg/kg; Sigma-Aldrich) dissolved in saline^42^,^45^, to ablate existing OSNs. Mice were perfused with fixative 7 (5 mice), 9 (5 mice), 13 (3 mice), 14 (6 mice), 19 (2 mice), or 21 (1 mouse) days following methimazole administration (Fig. 6b, and Supplementary Table 2).

### Optical imaging of spH signals

Olfactory sensory axon terminal activities were detected from heterozygous OMP-spH knock-in mice using a Nikon A1R confocal laser scanning microscope system attached to an upright ECLIPSE FN1 microscope (Nikon Corp., Tokyo, Japan)^74^. The fluorescent signal was detected using a 488 nm excitation laser and a 525/50 bandpass emission filter. Sequential images of the OB were acquired at two frames per s for at least 10 min. Off-line analysis was performed with ImageJ software (NIH) and its plug-in. Photobleaching was corrected by subtracting fluorescent changes observed in non-responsive glomeruli. Averaged fluorescence during 5 s (10 images) was expressed as one bin (onset, average ± SD; duration, average) in order to calculate significant increases in spH signals by one time administration of PST. SpH responses were calculated as ΔF/F_0_ = (F − F_0_)/F_0_, where F_0_ is the average baseline fluorescence (20 images) over the 10 s prior to PST administration. Excitatory spH responses were defined as those showing a significant increase in a bin (ten images) relative to the immediately preceding bin (ten images, Mann-Whitney *U-*test; p < 0.05 was considered statistically significant). If the responses were statistically significant, then this point was defined as the response onset. We determined the onset time, defined by the bin number after PST administration with the first significant increase in neural response. We also determined the peak neural response and the duration, defined as the number of bins from the first bin to that in which the spH returned to 50% of the peak responses (i.e., the time to 50% reduction in peak response). For the generation of pseudo-color images representing the relative increase in response after PST administration, we subtracted the averaged image during the 10 s before PST administration from the averaged image during the 10 s including the strongest spH signal after PST administration, and the resulting values were expressed as ΔF. All spH signals were scaled to the maximum as indicated in the heat map to the right of each pseudo-color image.

For comparing the averaged spH signal intensities between the open and occluded OB, a much larger region of interest than that used for the analysis of individual glomeruli was selected (Fig. 5d). The median intensity (0–100%) was calculated from the intensity histogram of each open and occluded OB using ImageJ and compared (nine open OBs and nine contralateral occluded OBs, Fig. 5c,d).

Averaged fluorescence during 5 s (10 images) was expressed as one bin (onset, average ± SD; duration, average).

For traces of the averaged spH signal intensities (Fig. 5e), averaged fluorescence during 5 s (one bin) of the occluded side were divided by those of the open side, and then their values were traced as relative spH signal intensity over 600 s.

### Immunohistochemistry

Some mice were intracardially perfused with 4% paraformaldehyde in 0.1 M phosphate buffer and decapitated, and the head was post-fixed for 24 h in the same fixative. The nasal tissues, including the OE, were decalcified using 10% EDTA solution, pH 7.0, and embedded in paraffin. Coronal sections (4 μm thick) were cut and mounted on silane-coated slides. Deparaffinized sections were autoclaved for 10 min in Target Retrieval Solution (S1700; Dako) for antigen retrieval. Immunohistochemistry was performed using anti-OMP (goat polyclonal, 1:4,000 dilution; Wako Chemicals). The immunoreaction was detected using the Histofine Simple Stain MAX-PO secondary antibody system (Nichirei) for goat anti-OMP.

### Histological analyses

Unilateral OE analyses were performed on the following three areas: the concha bullosa, upper nasal septum, and lower nasal septum. Three coronal sections located between the caudal OE region and the caudal end of the silicone tube were examined to minimize effects resulting from direct contact with the silicon tube^42^ (9 areas per mouse). Sections were cut at 500 μm intervals. The olfactory neuroepithelium contains three major cell types: OSNs, supporting cells, and basal stem cells. We defined supporting cells as columnar cells located more apically in the OE and basal cells as rectangular cells lying on the lamina propria. The remaining cells were defined as OSNs. The number of OSNs labeled by anti-OMP antibodies was quantitatively analyzed using sections with single immunostaining for each antigen and counterstained with hematoxylin. Cells with an immunostaining intensity exceeding two SDs of the mean background intensity for the connective tissue under the lamina propria were considered OMP-positive. The mean number of OMP-positive cells per 100 μm length of OE was then calculated for each mouse.

### Statistical analyses

Statistical analyses were performed using the Mann-Whitney *U-*test for the data in Figs 2d,e, 4d,f,l,m and 5d,g,h,l,m, as well as for the data in Supplementary Figs 1a, 1b, 2b, 3e, 3f, 3g, 5d, 5e, and 5f. Two-way ANOVA was used for the data in Figs 2i,j, 4n,o and 6c,d, as well as for the data in Supplementary Fig. 3h,i, and the significance tests of non-zero values of Pearson’s correlation coefficient were used for the data in Fig. 6o,p. All values are expressed as mean ± standard deviation (SD). A p-value of < 0.05 was considered statistically significant.

### Study approval

Human research was conducted according to the Helsinki Declaration and the Japanese Ethical Guidelines for Epidemiology Research. The protocol was approved by the ethics committee of Jikei University School of Medicine. Before clinical examination and ESS, informed consent was obtained from all patients. All animal studies were approved by the Experimental Animal Research Committee at the University of Tokyo, and the animal study methods were carried out in accordance with the approved guidelines.

## Additional Information

**How to cite this article**: Kikuta, S. *et al*. Longer latency of sensory response to intravenous odor injection predicts olfactory neural disorder. *Sci. Rep.* **6**, 35361; doi: 10.1038/srep35361 (2016).

## Supplementary Material

Supplementary Information

Supplementary Movie 1

Supplementary Movie 2

## Figures and Tables

**Figure 1:**
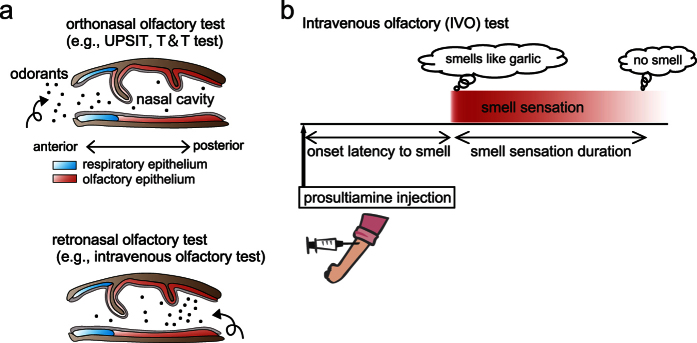
Two types of olfactory function tests. (**a**) Two olfactory function tests distinguished by stimulus route: orthonasal (upper) and retronasal (lower). Sagittal views of the nasal cavity are shown (left, anterior; right, posterior). UPSIT, University of Pennsylvania Smell Identification Test; T & T olfactometer test, an orthonasal olfactory test used widely in Japan. (**b**) Human intravenous olfactory (IVO) test. In the IVO test, two factors, the onset latency from the injection of prosultiamine to the recognition of smell and the duration between the recognition and disappearance of smell, are measured to detect olfactory disorders.

**Table 1:** Univariable and multivariable measures of pre-operative factors associated with post-operative olfactory outcome.

Variable	N = 253	Mean (pre)	Mean (post)	Univariable OR (95% CI)	p-value	Multivariable OR (95% CI)	p-value
age (years)		45.4 ± 13.89		1.01 (0.99–1.03)	0.26		
sex	male 179 (71%)			0.85 (0.49–1.49)	0.58		
	female 74 (29%)						
primary or revision	primary 198 (78%)			0.95 (0.52–1.76)	0.88		
	revision 55 (22%)						
CRS with/without polyp	with polyp 186 (74%)			1.22 (0.7–2.15)	0.49		
	without polyp 67 (26%)						
Likart scale score (sense of smell)		3.8 ± 1.9	1.4 ± 1.5				
Likart scale score (nasal discharge)		2.8 ± 1.7	0.8 ± 1.1	0.99 (0.87–1.14)	0.92		
Likart scale score (nasal obstruction)		3.8 ± 1.6	0.7 ± 0.9	0.98 (0.85–1.12)	0.78		
IVO (relative onset latency)		1.8 ± 0.8		2.34 (1.53–3.56)	0.0001	2.22 (1.43–3.44)	0.0004
IVO (relative duration)		0.9 ± 0.2		0.69 (0.2–2.3)	0.55		
T & T olfactometry score (detection)		3.5 ± 1.9		1.11 (0.97–1.26)	0.14	1.1 (0.83–1.44)	0.53
T & T olfactometry score (recognition)		4.1 ± 1.5		1.11 (0.94–1.31)	0.2	0.94 (0.66–1.34)	0.74
CT score (total)		10.2 ± 5.5		1.01 (0.96–1.05)	0.83		
maxillary sinus		2.0 ± 0.9		1.05 (0.79–1.41)	0.73		
anterior ethmoidal sinus		2.3 ± 1.0		0.95 (0.75–1.22)	0.7		
posterior ethmoidal sinus		1.9 ± 1.1		1.11 (0.89–1.39)	0.37		
sphenoid sinus		1.1 ± 1.3		1.13 (0.93–1.38)	0.23		
frontal sinus		1.7 ± 1.4		0.97 (0.81–1.17)	0.78		
ostiomeatal complex		1.1 ± 1.6		0.95 (0.82–1.2)	0.56		
IgE		246.7 ± 437.8		1.0 (0.99–1.0)	0.91		
% eosinophils		6.2 ± 4.7		1.02 (0.96–1.07)	0.56		
number of eosinophils (/μL)		371.0 ± 325.8		1.0 (0.99–1.0)	0.61		
allergic rhinitis	yes 121 (48%)			1.05 (0.63–1.74)	0.85		
	no 132 (52%)						
asthma	yes 41 (16%)			1.29 (0.64–2.6)	0.48		
	no 212 (84%)						
diabetes mellitus	yes 14 (6%)			0.85 (0.29–2.53)	0.77		
	no 239 (94%)						
smoking	current smoke 61 (24%)			0.94 (0.52–1.68)	0.82		
	non-smoker 192 (76%)						
	brinkman index	449.5 ± 505.1		1.0 (1.0–1.001)	0.2	1.0 (1.0–1.001)	0.48
