# Supplementary material for: Longer latency of sensory response to intravenous odor injection predicts olfactory neural disorder
Source: Sci Rep. 2016 Oct 13;6:35361. doi: 10.1038/srep35361 (PMC5062120; doi:10.1038/srep35361)
Supplement: Supplementary Information [file srep35361-s1.pdf]

Longer latency of sensory response to intravenous odor injection predicts olfactory neural disorder

Shu Kikuta*, Yu Matsumoto*, Akihito Kuboki, Tsuguhisa Nakayama, Daiya Asaka, Nobuyoshi Otori, Hiromi Kojima, Takashi Sakamoto, Kashio Akinori, Kaori Kanaya, Rumi Ueha, Ryoji Kagoya, Hironobu Nishijima, Makiko Toma-Hirano, Yayoi Kikkawa, Kenji Kondo, Koichi Tsunoda, Tempei Miyaji, Takuhiro Yamaguchi, Kazunori Kataoka, Kensaku Mori, and Tatsuya Yamasoba

* These authors contributed equally to this work

Corresponding author: Shu Kikuta

Supplementary information

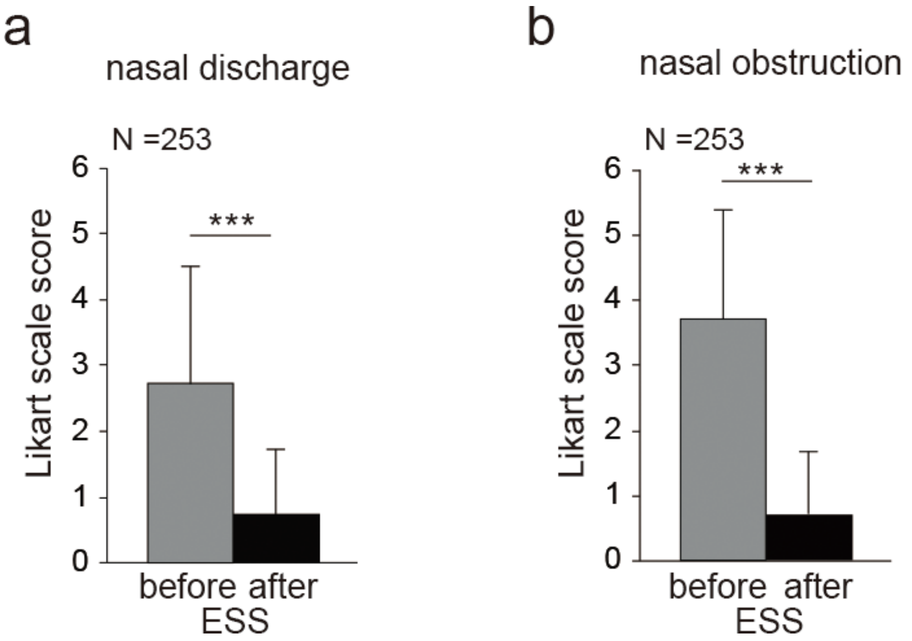

Supplementary Figure 1. Endoscopic sinus surgery (ESS) improves Likart scale scores for nasal discharge and obstruction

(**a, b**) Comparisons of Likart scale scores for nasal discharge (**a**) and nasal obstruction (**b**) before and after ESS. Statistical comparisons using Mann–Whitney *U*-test (a and b). ***$p < 0.001$. All values are mean ± SD.

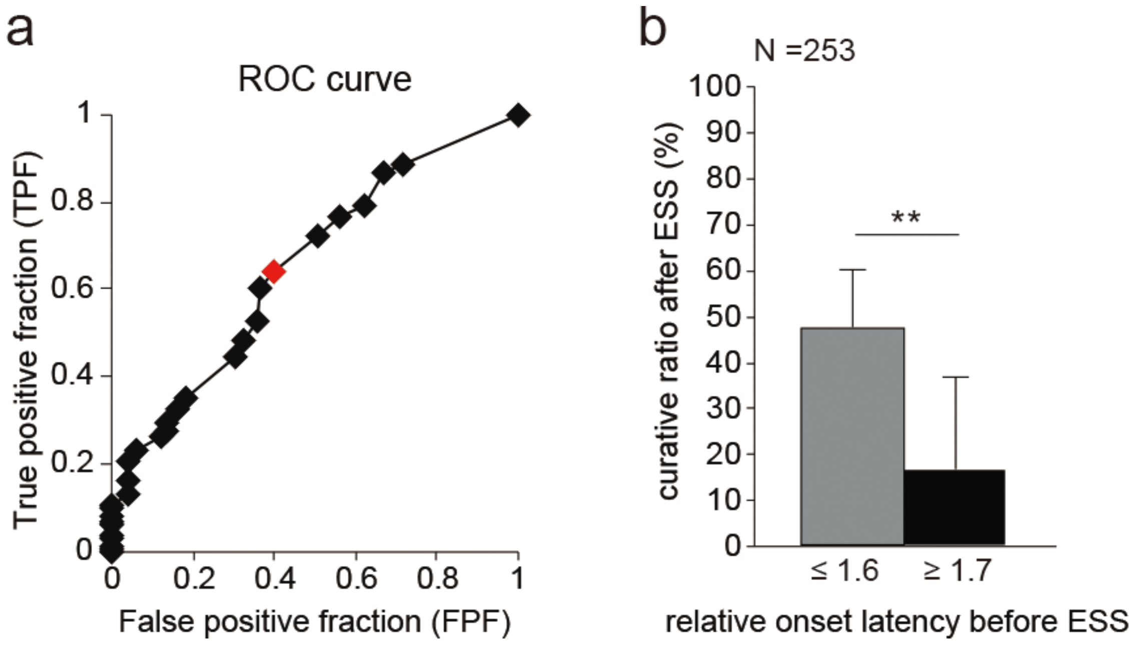

Supplementary Figure 2. Receiver operator characteristic (ROC) curve analysis

(**a**) ROC curves of the modeling dataset. The curve was created by plotting the true positive fraction (TPF, y-axis) known as sensitivity against the false positive fraction (FPF, x-axis) known as 1- specificity at various threshold settings. The red dot indicates the cut off value as the point on the curve closest to the upper left corner in the ROC (sensitivity;64%, specificity;60%).

(**b**) Comparison of the curative ratio following ESS in the optimum cut off value of 1.6. Statistical comparisons using Mann–Whitney *U*-test. **p < 0.01. All values are mean ± SD.

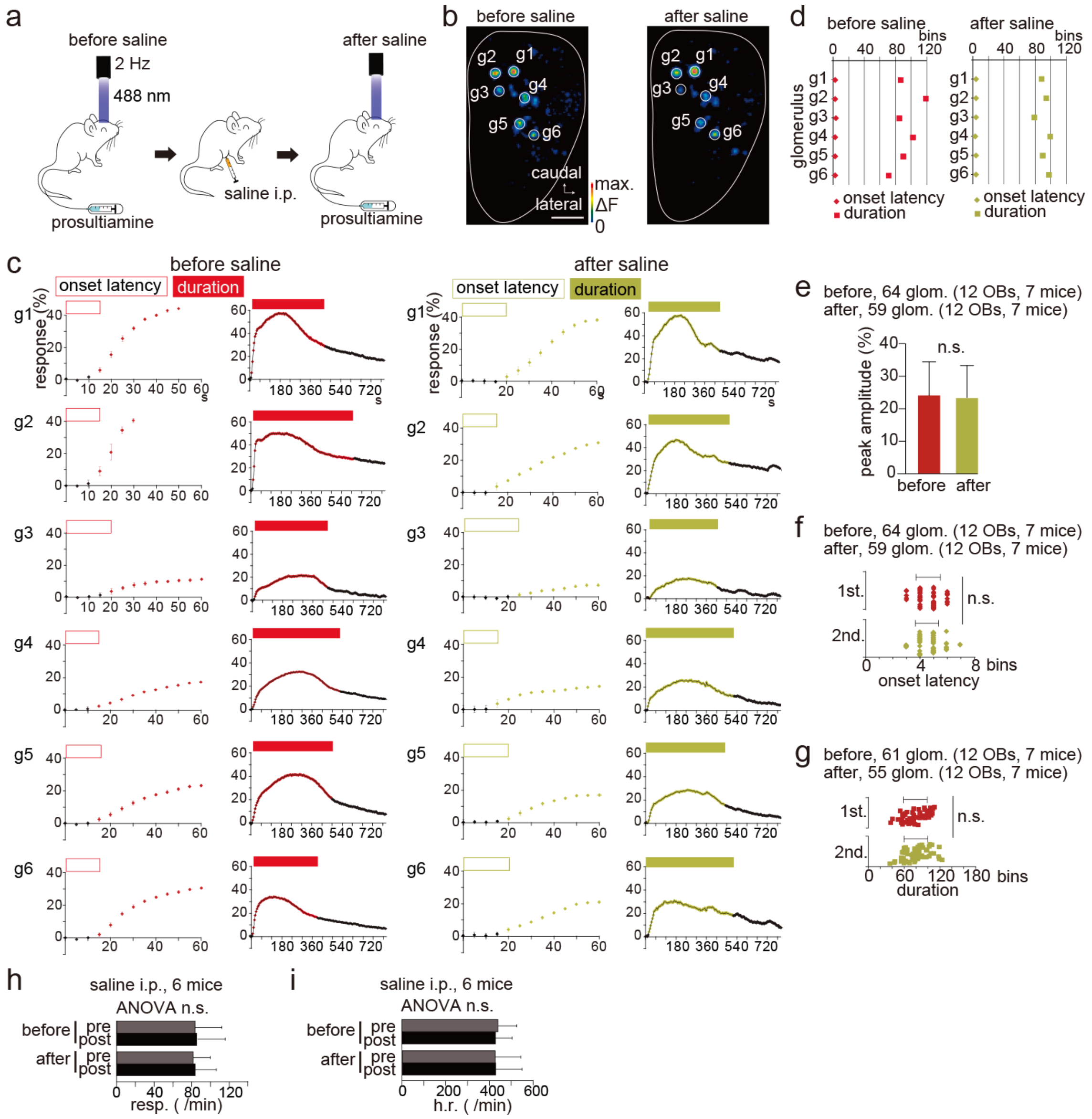

Supplementary Figure 3. No significant difference in neural response delay and duration between before and after intravenous prosultiamine (PST) injections

(**a**) Experimental protocol for consecutive PST injections. PST-induced synaptopHluorin (spH) signals were recorded before (left) and after i.p. saline administration (right).

(**b**) Images of the olfactory bulb (OB) dorsal surface before (left) and after saline administration

(right) from glomeruli g1–g6. Scale bar, 500 μm.

(**c**) Two sequential PST-induced spH responses from individual glomeruli (g1–g6). Before saline administration (left) and after saline administration (right). Open rectangle, onset latency; filled rectangle, duration.

(**d**) Summary of onset latency and duration in each glomerulus shown in (**b**). Red diamond, onset latency; red square, duration.

(**e**) Comparison of peak amplitude between before and after saline administration. There are no significant differences. Red, before saline administration; yellow, after saline administration. glom, glomerulus.

(**f, g**) Comparison of onset latency (f) and duration (g) before and after saline administration. No significant differences in onset latency and duration are detected.

(**h, i**) Comparison of respiration (resp., **h**) and heart rate (h.r., **i**) before and after saline administration. Each recording includes the period of pre- (pre) and post-response (post). Statistical comparisons were done using Mann–Whitney *U*-test (**e, f,** and **g**) and two-way ANOVA (**h** and **i**). n.s., no significant difference. All values are mean ± SD.

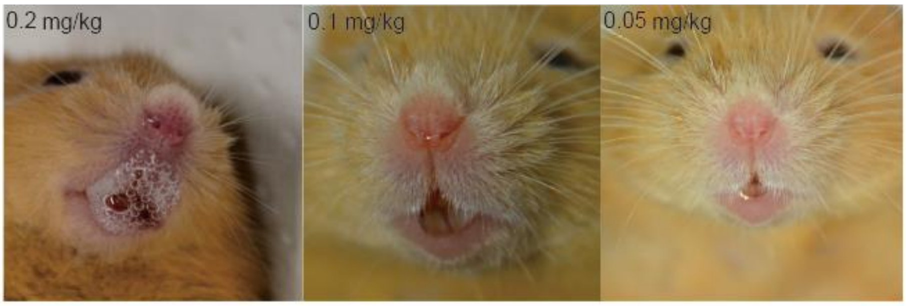

nasal discharge	+++	++	+
saliva	+++	++	+

Supplementary Figure 4. Pilocarpine increases nasal discharge and saliva

Each photograph shows the frontal view of a mouse after the administration of a different pilocarpine concentration (0.2, 0.1, and 0.05 mg/kg). +++, prominent increase in nasal discharge and saliva; +, little increase; ++, intermediate increase.

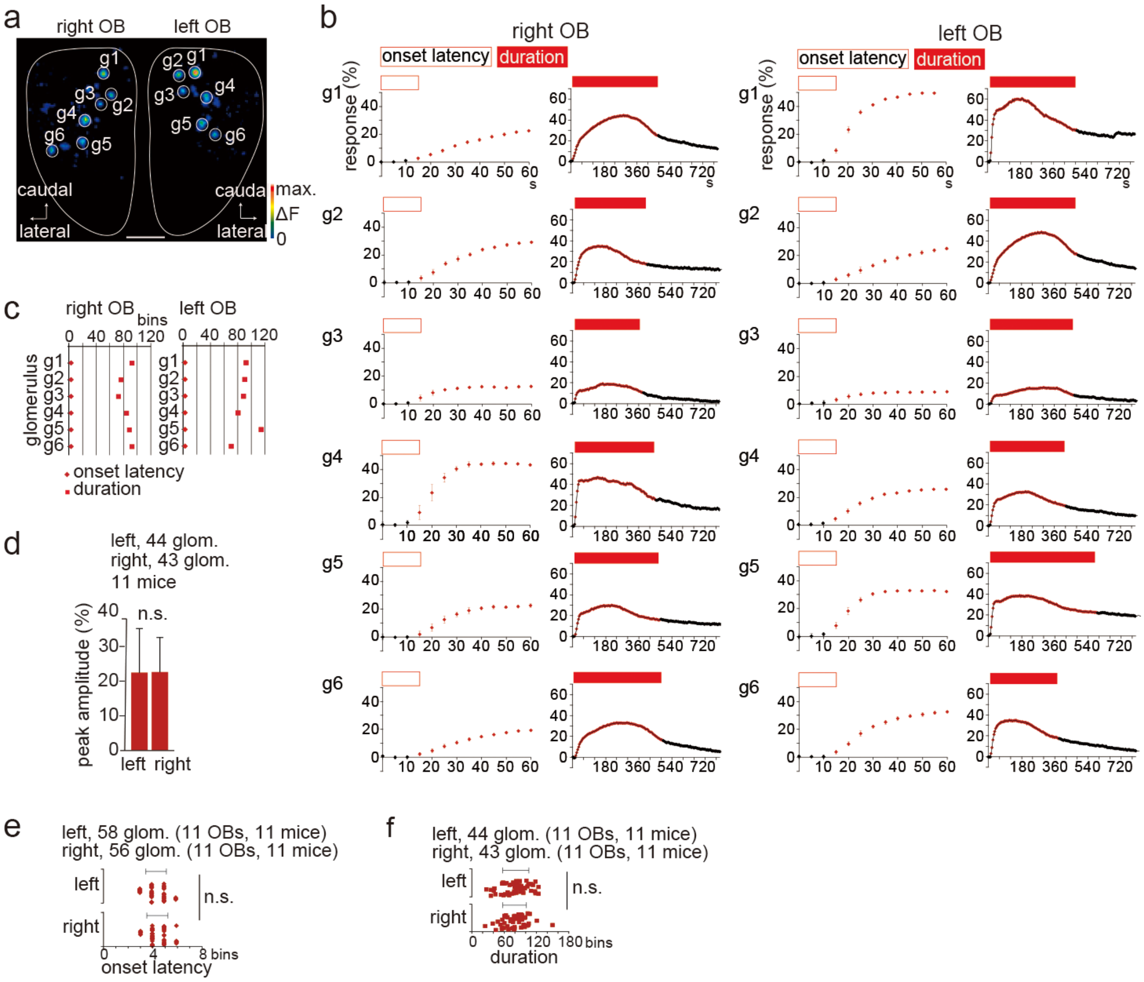

Supplementary Figure 5. No significant differences in onset time and duration between right and left olfactory bulbs following prosultiamine (PST) injection

(**a**) Images of the right and left OE dorsal surfaces during PTS-induced synaptopHluorin (spH) responses. g1–g6 are individual glomeruli on each side. OB, olfactory bulb. Scale bar, 500 µm.

(**b**) PST-induced spH responses of individual glomeruli (g1–g6) in both right and left OBs.

(**c**) Summary of onset latency and duration for each glomerulus shown in (**a**). Red diamond, onset latency; red square, duration.

(**d**) Comparison of peak amplitude between right and left OBs. Significant differences are not

detected. glom., glomerulus.

(**e, f**) Comparison of onset latency and duration between right and left OBs. Significant differences are not detected. Statistical comparisons by Mann–Whitney *U*-test (**d, e,** and **f**). n.s., no significant difference. All values are mean ± SD.

Condition	Mice	Side	Response ※1	Histology	Related figure	Note
Normal	#1	rt	Yes (PST)	Yes	Figs. 2d, e, 3e, 6c, d, o, p	
		lt	Yes (PST)	Yes	Figs. 2d, e, 3e, 6c, d, o, p, 6e, f, g	
	#2	rt	Yes (PST)	Yes	Figs. 2d, e, 3e, 6c, d, o, p, Supplementary Fig. 5a, b, c, d, e, f	
		lt	Yes (PST)	Yes	Figs. 2d, e, 3e, 6c, d, o, p, Supplementary Fig. 5a, b, c, d, e, f,	
	#3	rt	Yes (PST)	Yes	Figs. 2d, e, 3e, 6c, d, o, p	
		lt	Yes (PST)	Yes	Figs. 2d, e, 3e, 6c, d, o, p	
	#4	rt	Yes (PST)	Yes	Figs. 2d, e, 3e, 6c, d, o, p	
		lt	Yes (PST)	Yes	Figs. 2d, e, 3e, 6c, d, o, p	
	#5	rt	Yes (PST)	Yes	Figs. 2d, e, 3e, 6c, d, o, p	
		lt	Yes (PST)	Yes	Figs. 2d, e, 3e, 6c, d, o, p	
	#6	rt	Yes (PST)	No	Figs. 2d, e, 3e, 6c, d	
		lt	Yes (PST)	No	Figs. 2b, c, d, e, 3e, 6c, d,	
	#7	lt	Yes (PST)	No	Fig. 3a, b, c, e	
	#8	rt	Yes (PST)	No	Figs. 2d, e, 3e, 6c, d, Supplementary Fig. 5d, e, f	
		lt	Yes (PST)	No	Figs. 2d, e, 3e, 6c, d, Supplementary Fig. 5d, e, f	
	#9	rt	No (saline)	No	Fig. 2c	
	#10	rt	No (saline)	No		
	#11	rt	No (saline)	No		
		lt	No (saline)	No		
Saline administration	#1	rt	Yes (PST)	Yes	Figs. 3e, 4d, Supplementary Fig. 3e, f, g, h, i	※2
		lt	Yes (PST)	No	Fig. 3e, Supplementary Fig. 3b, c, d, e, f, g, h, i	※2
	#2	rt	Yes (PST)	Yes	Figs. 3e, 4d, Supplementary Fig. 3e, f, g, h, i, Supplementary Fig. 5d, e, f	※2
		lt	Yes (PST)	No	Fig. 3e, Supplementary Fig. 3e, f, g, h, i, Supplementary Fig. 5d, e, f	※2
	#3	rt	Yes (PST)	No	Fig. 3e, Supplementary Fig. 3e, f, g, h, i, Supplementary Fig. 5d, e, f	※2
		lt	Yes (PST)	No	Fig. 3e, Supplementary Fig. 3e, f, g, h, i, Supplementary Fig. 5d, e, f	※2
	#4	lt	Yes (PST)	No	Fig. 3e, Supplementary Fig. 3e, f, g	※2
	#5	lt	Yes (PST)	No	Fig. 3e, Supplementary Fig. 3e, f, g, h, i	※2
	#6	rt	Yes (PST)	No	Supplementary Fig. 3e, f, g, h, i, Supplementary Fig. 5d, e, f	※2
		lt	Yes (PST)	No	Supplementary Fig. 3e, f, g, h, i, Supplementary Fig. 5d, e, f	※2
	#7	rt	Yes (PST)	No	Fig. 3e, Supplementary Fig. 3e, f, g, h, i, Supplementary Fig. 5d, e, f	※2
		lt	Yes (PST)	No	Fig. 3e, Supplementary Fig. 3e, f, g, h, i, Supplementary Fig. 5d, e, f	※2
	#8	rt	n.d.	Yes	Fig. 4d	
		lt	n.d.	Yes	Fig. 4d	
	#9	rt	n.d.	Yes	Fig. 4d	
		lt	n.d.	Yes	Fig. 4d	
Tracheotomy	#1	lt	No (PST)	No		
	#2	lt	No (PST)	No	Fig. 2g, h, i, j	
	#3	rt	No (PST)	No	Fig. 2i, j	
	#4	rt	No (PST)	No	Fig. 2i, j	
	#5	rt	No (PST)	No		

Supplementary Table 1. Number of animals in normal conditions.

n.d., not determined.

※1; response to stimulation with PST or saline

※2; The results in Fig. 3 and Supplementary Fig. 5 were obtained from the first trial before saline administration.

Condition	Mice	Days following injury	Side	Response ※1	Histology	Related figure	Note
Pirocalpine administration	#1		rt	No	No	Fig. 4e	
			lt	No	No		
	#2		rt	No	No	Fig. 4e	
	#3		rt	No	No	Fig. 4e	
	#4		lt	No	No	Fig. 4e	
	#5		rt	Yes	No	Figs. 3e, 4e, l, m, n, o, Supplementary Fig. 5d, e, f	※2
	#6		lt	Yes	No	Figs. 3e, 4e, l, m, n, o, Fig. 4g, h, i, j, k, Supplementary Fig. 5d, e, f	※2
	#7		rt	Yes	Yes	Figs. 3e, 4d, e, l, m, n, o	※2
	#8		rt	Yes	No	Fig. 4e, l, m, Supplementary Fig. 5d, e, f	※2
			lt	Yes	No	Fig. 4e, l, m, Supplementary Fig. 5d, e, f	※2
	#9		rt	Yes	No	Fig. 4e, l, m, Supplementary Fig. 5d, e, f	※2
			lt	Yes	No	Fig. 4e, l, m, Supplementary Fig. 5d, e, f	※2
	#10		rt	Yes	No	Fig. 4e, l, m, n, o, Supplementary Fig. 5d, e, f	※2
			lt	Yes	No	Fig. 4e, l, m, n, o, Supplementary Fig. 5d, e, f	※2
	#11		rt	Yes	No	Fig. 4e, l, m, n, o, Supplementary Fig. 5d, e, f	※2
			lt	Yes	No	Fig. 4e, l, m, n, o, Supplementary Fig. 5d, e, f	※2
	#12		rt	No	No	Fig. 4e	
	#13		rt	No	No	Fig. 4e	
	#14		rt	n.d.	Yes	Fig. 4d	
	#15		rt	n.d.	No	Supplementary Fig. 4	
	#16		rt	n.d.	No	Supplementary Fig. 4	
	#17		rt	n.d.	No	Supplementary Fig. 4	
	#18		rt	n.d.	Yes	Fig. 4d	
			lt	n.d.	Yes	Fig. 4d	
	#19		rt	n.d.	Yes	Fig. 4d	
			lt	n.d.	Yes	Fig. 4d	
	#20		rt	n.d.	Yes	Fig. 4d	
			lt	n.d.	Yes	Fig. 4d	
Nostril occlusion	#1			Yes	No	Fig. 5b, d, h, i, j, k, l, m	
	#2			Yes	No	Fig. 5d, h, l, m	
	#3			Yes	No	Fig. 5d, h, l, m	
	#4			Yes	Yes	Fig. 5d, g, h, l, m	
	#5			Yes	Yes	Fig. 5d, g, h, l, m	
	#6			Yes	No	Fig. 5d, h, l, m	
	#7			Yes	Yes	Fig. 5d, h, l, m	
	#8			Yes	No	Fig. 5d, h, l, m	
	#9			Yes	No	Fig. 5d, h, l, m	
	#10			Yes	No	Fig. 5e	
	#11			Yes	No	Fig. 5e	
	#12			Yes	No	Fig. 5e	
	#13			n.d.	Yes	Fig. 5g	
	#14			n.d.	Yes	Fig. 5g	
Methimazole administration	#1	7	rt	No	No	Fig. 6b	
			lt	No	No	Fig. 6b	
	#2	7	rt	No	No	Fig. 6b	
			lt	No	No	Fig. 6b	
	#3	7	rt	No	No	Fig. 6b	
			lt	No	No	Fig. 6b	
	#4	7	rt	No	No	Fig. 6b	
			lt	No	No	Fig. 6b	
	#5	7	rt	No	No	Fig. 6b	
			lt	No	No	Fig. 6b	
	#6	9	rt	No	No	Fig. 6b	
			lt	No	No	Fig. 6b	
	#7	9	rt	No	No	Fig. 6b	
			lt	No	No	Fig. 6b	
	#8	9	rt	No	No	Fig. 6b	
			lt	No	No	Fig. 6b	
	#9	9	rt	No	No	Fig. 6b	
			lt	No	No	Fig. 6b	
	#10	9	rt	No	No	Fig. 6b	
			lt	No	No	Fig. 6b	
	#11	13	rt	Yes	Yes	Fig. 6b, o, p	
			lt	Yes	Yes	Fig. 6b, o, p	
	#12	13	rt	No	No	Fig. 6b	
			lt	No	No	Fig. 6b	
	#13	13	lt	Yes	Yes	Fig. 6b, c, d, o, p	
	#14	14	rt	Yes	Yes	Fig. 6b, c, d, o, p	
			lt	Yes	Yes	Fig. 6b, c, d, k, l, m, o, p	
	#15	14	rt	Yes	Yes	Fig. 6b, c, d, o, p	
			lt	Yes	Yes	Fig. 6b, c, d, o, p	
	#16	14	rt	No	No	Fig. 6b	
			lt	No	No	Fig. 6b	
	#17	14	rt	Yes	Yes	Fig. 6b, c, d, o	※3
			lt	Yes	Yes	Fig. 6b, c, d, o	※3
	#18	14	rt	No	No	Fig. 6b	
			lt	No	No	Fig. 6b	
	#19	14	rt	Yes	Yes	Fig. 6b, c, d, o, p	
			lt	Yes	Yes	Fig. 6b, c, d, o, p	
	#20	19	rt	No	No	Fig. 6b	
			lt	No	No	Fig. 6b	
	#21	19	rt	Yes	Yes	Fig. 6b, c, d, o, p	
	#22	21	rt	Yes	Yes	Fig. 6b, o, p	
			lt	Yes	Yes	Fig. 6b, h, i, j, o, p	

Supplementary Table 2. Number of animals in pathological conditions.

n.d., not determined.

※1; response to stimulation with PST

※2; The results in Fig. 3 and Supplementary Fig. 5 were obtained from the first trial before pilocarpine administration.

※3; In this mouse, response duration was not determined.

Supplementary movie legends

Supplementary movie 1: SynaptopHluorin responses of individual glomeruli to stimulation with prosultiamine

The dorsal surface of the olfactory bulb was exposed, and the activity of individual glomeruli was recorded using confocal microscopy. Arrowheads show representative responsive glomeruli. Individual glomeruli exhibit neural responses almost simultaneously. Acq. Time, acquisition time.

Supplementary movie 2: SynaptopHluorin responses of individual glomeruli to prosultiamine in both open and occluded olfactory bulbs (OBs)

Arrowheads show representative responsive glomeruli. Individual glomeruli in both open (right side) and occluded OBs (left side) exhibit neural responses almost simultaneously. Acq. Time, acquisition time.
